# Part I of “Navigating the transition”: early-career working conditions, psychosocial demands, and job satisfaction among German, Austrian, and Swiss veterinarians – a first-year survey

**DOI:** 10.1186/s12917-026-05923-9

**Published:** 2026-09-21

**Authors:** Amelie Wetter, Timo Lorenz, Marcus G. Doherr, Katharina Charlotte Jensen

**Affiliations:** 1 Institute for Veterinary Epidemiology and Biostatistics, School for Veterinary Medicine, Freie Universität Berlin, Berlin, Germany; 2 MSB Medical School Berlin, Hochschule für Gesundheit und Medizin, Berlin, Germany

**Keywords:** Survey, Mental health, Occupational psychology, Veterinary medicine, Veterinarians

## Abstract

**Background:**

Starting a career in veterinary medicine represents a major life transition, accompanied by substantial professional and personal challenges. The study aimed to assess *job satisfaction*, psychosocial demands, and working conditions of veterinarians during their first year in their profession, and to identify the factors associated with *commitment* and *job satisfaction*.

Early-career veterinarians in Germany, Austria, and Switzerland were surveyed using an online questionnaire that included the Copenhagen Psychosocial Questionnaire III. Multifactorial regression models were calculated to reveal the associations of (a) demographics and working conditions and (b) psychosocial demands with commitment and job satisfaction.

**Results:**

Responses from 196 participants were included in the analysis. The majority of participants (n=118; 76%) were engaged in clinical practice, predominantly in the small animal sector. Half of the respondents already participated in emergency services, and another 17% planned to do so in the near future. Most respondents reported overtime work. Compared with the German COPSOQ standard sample, early-career veterinarians reported lower levels of role conflicts and higher levels of job satisfaction, commitment to the workplace, and sense of community at work. At the same time, dissolution (work-life boundary blurring) was higher than in the German standard sample. Job satisfaction and commitment were primarily associated with the availability of a supervising veterinarian, perceived quality of leadership, support at work, and feedback. Interestingly, neither quantitative demands (amount of work related to time) nor emotional demands (emotionally distressing work situations) were significantly associated with commitment to the workplace or job satisfaction.

**Conclusion:**

The results indicate that early-career veterinarians are highly motivated and satisfied with their work but strongly benefit from adequate supervision, feedback, and organizational support.

**Supplementary Information:**

The online version contains supplementary material available at https://doi.org/10.1186/s12917-026-05923-9.

## Background

Mental health in veterinary medicine is an increasingly recognized and important topic [1]. Several international studies showed that veterinarians have higher rates of mental ill-health, including depression, anxiety disorders, burnout, work-related stress, and higher suicide rates compared to the general population [2, 3]. This was also shown for veterinarians in Germany and Austria [4, 5]. The risk factors contributing to mental ill-health have also been examined in numerous studies. Working conditions, especially working hours, and ethical challenges are major sources of stress [2, 3]. Veterinarians often work overtime, on call and in some cases night shifts [6, 7], which may lead to sleep deprivation [8]. However, individual factors such as overcommitment and personality also seem to play a crucial role [9, 10]. Some studies exploring poor mental health in veterinarians reported higher levels of stress among in young and/or inexperienced veterinarians [1, 11–13]. Moreover, many young veterinarians associate their entry into the profession with uncertainty and stress [14]. In light of the current shortage of skilled workers [15], this anticipation could possibly hinder graduates from entering veterinary medicine and thereby exacerbate the shortage. At the same time, the veterinary profession can be a rich source of personal fulfillment [16, 17]. Helping animals and people, and thereby contributing to society, creates feelings of meaningfulness and well-being among veterinarians [17]. Collaborating with colleagues or supervisors and a good atmosphere at the workplace are additional factors that contribute *to* job satisfaction and a high commitment to the workplace [18–20]. However, when starting a veterinary career, people might feel alone or insecure because they have not yet become part of the team. Studies from other professions showed that, as professionals navigate from orientation to settling in, the professional fit, relationships, and issues of competence and confidence indeed change [21]. For career starters in their first year, self-efficacy and professional support positively influence job satisfaction [22]. On the other hand, a lack of administrative and collegial support, along with time pressure, leads to dissatisfaction [23]. There are qualitative studies focusing on the demands regarding career entry into veterinary medicine. These studies revealed relevant factors, such as the importance of informal support and professional identity, and the balancing of self-doubt [24, 25]. Other studies dealing with the transition focus primarily on the skills needed [26] or address mostly general aspects of the work situation without establishing a direct link between working conditions and psychosocial stress [27]. This makes it difficult to assess job satisfaction and psychosocial demands, and to compare them with those of other professions or veterinarians in later stages of their careers. The burden (or fulfillment) that early-career veterinarians carry cannot be deduced from these studies. This study aims at filling this gap by assessing working conditions, psychosocial demands, and mental health of veterinarians entering the profession. Validated instruments were used allowing comparisons to reference populations. By identifying risk factors, recommendations can be derived and evidence-based support can be provided to those entering the veterinary profession and to those who lead veterinarians in their early careers. In this first publication, we aimed to shed light on the working conditions and associated psychosocial demands of veterinarians in their first year, and to examine their associations with the *commitment to the workplace* and *job satisfaction*. We hypothesized that.

1. High levels of demands, *dissolution of boundaries*, and *role conflicts* are present in early-career veterinarians compared to the COPSOQ reference population,
2. Lower *job satisfaction* is associated with higher demands and poorer working conditions, such as overtime, participation in emergency services, and lack of supervision/mentorship,
3. *Commitment to the workplace* is associated with working conditions as well as the assessment of social relationships within the workplace, like working atmosphere.

On this basis, specific recommendations for action can be derived. The findings of this work should help to strengthen supportive working conditions in a targeted manner and enable young veterinarians to have a successful, satisfied, and sustainable start to their careers.

## Materials and methods

### Survey

The study was approved by the Chair of the Commission for Research Ethics of the Free University of Berlin (no. ZEA24030). It was conducted in compliance with German and European data protection regulations. The participants had to give their informed consent on the landing page of the survey.

The target group for the survey was veterinarians who graduated in 2024 or who had worked as veterinarians for less than 12 months. Only those participants working in Germany, Austria, or Switzerland were included.

Based on hypotheses and literature research, a questionnaire was created containing questions on demographics and work characteristics, as well as sections from other validated questionnaire-based assessments: the Copenhagen Psychosocial Questionnaire III [28], Oldenburg Burnout Inventory [29], Patient Health Questionnaire − 4 [30], and Psychological Capital Inventory [31].

The questionnaire, including all components, was implemented in LimeSurvey (LimeSurvey Cloud version 6.6.8). Five veterinarians in different stages of their professional lives tested it in September 2024. Feedback was provided by email and via additional free-text fields. The comments resulted in minor changes to the original version.

The survey was disseminated via social media, such as Instagram and WhatsApp groups, and by the Association of Independent Pet Clinics (Verbund Unabhängiger Kleintierkliniken e.V.). It was open for completion from the beginning of October to the end of December 2024.

### Questionnaire structure

The first section of the questionnaire is attached as supplementary material in the original language (German; S1). An overview of sections, implemented instruments, and the number of items is displayed in Table 1. The results on burnout, anxiety, and psychological capital will be included in a second publication.

**Table 1:** Overview of the questionnaire used to assess the working conditions, psychosocial demands, mental health, and psychological capital of early-career veterinarians in Germany, Austria, and Switzerland (2024)

Section	Domains	Instrument	Number of Items	Response Formats	Response scales	Examples for items	References
1	Survey Code, demographics, employment status, living situation, job domain, working hours, overtime, professional experience	Self-developed	54	Single-choice, multiple-choice, free text			
2	Psychosocial work environment and job satisfaction	Copenhagen Psychosocial Questionnaire Version III (German standard version)	38	Likert scales	Different scales like: To a very large extent (100); To a large extent (75); Somewhat (50); To a small extent (25); To a very small extent (0)	*Emotional demands*: Is your work emotionally demanding? *Job Satisfaction*: Regarding your work in general. How pleased are you with the way your abilities are used?	[28, 32, 33]
3	Burnout (exhaustion, disengagement)	Oldenburg Burnout Inventory (OLBI)	16	Likert scales	1 = strongly agree 2 = agree 3 = disagree 4 = strongly disagree	I find myself speaking disparagingly about my workplace more and more often. After work, I often need longer periods of rest to recover.	[29]
4	Depressive symptoms and anxiety	Patient Health Questionnaire-4 (PHQ-4)	4	Likert scales	3 = Almost every day 2 = more than half of the days 1 = several days 0 = not at all	Little interest or pleasure in doing things Feeling nervous, anxious, or on edge	[30]
5	Psychological capital (hope, resilience, self-efficacy, optimism)	Psychological Capital Inventory (PSI-16)	16	Likert scales	1 = strongly disagree; 6 = strongly agree	*Optimism*: I look positively towards the future and look forward to upcoming experiences.	[31]
Total			128				

### Statistical analyses

The statistical analyses were carried out with IBM SPSS Statistics Version 29.0.2.0 [20].

### Modification of variables

Three quantitative variables were assigned to categories to ensure a compact overview. The question about the number of employees at the workplace was divided into three categories: <10, 10–30, and > 30. Information on actual working hours was collected in hours per week and categorized into < 40, 40, and > 40 h/week. Overtime was determined by the difference between the contractually agreed and actual working hours per week. These were then divided into the categories: 0 h (no overtime), > 0–10, > 10–20, and > 20 h per week.

The question about the veterinary school at which the course of study had been completed was summarized into a new variable. All veterinary schools not located in Germany were pooled into the category “universities outside of Germany” due to the small number of respective respondents. Responses to the question about the animal species that they mainly treated were also pooled. Most respondents worked mainly with small animals (dogs, cats, but also other companion animals as rabbits or guinea pigs), ruminants, or horses. All other animal species were summarized under the category “other species” due to the small number of respondents. The answers regarding emergency service were summarized as “yes” (already participating in emergency service) or “no” (not planned or not yet participating). The answers regarding a doctoral thesis were also summarized as “Yes” (“Yes, in context of my employment” and “Yes, in addition to my veterinary work”) and “No”.

For the multifactorial modeling, missing values for the COPSOQ items were imputed using multiple imputation in SPSS. If more than 7 data points were missing for a single participant, or if a participant did not answer a single item on a specific dimension, the data were not imputed, and the participant was excluded from the multifactorial analysis. The imputation was done using fully conditional specification (FCS) with linear regression. The total proportion of missing values for the COPSOQ was 1% (59 of 5548 datapoints). The dimensions were imputed individually. If all entries in a dimension were missing, they were not imputed but were excluded.

### Descriptive statistics

Descriptive analyses were carried out using frequencies, means, and standard deviations, as well as figures such as boxplots and scatterplots. The dimensions of the COPSOQ were evaluated according to the guideline and compared with the German reference population [28]. Moreover, Cronbach´s alpha was calculated to test the internal consistency of each dimension with more than one item.

Based on defined hypotheses, four multifactorial linear regression models were calculated for the dependent variables *commitment* and *job satisfaction*. In the first two models, demographic information (gender, relocation, practical experience, dissertation, and activity) as well as work characteristics (emergency service, fixed-term employment contract, supervising veterinarian, debts (e.g., student loan), and duration of current activity) were included. In the other two models, the associations between other psychosocial dimensions (*quantitative demands*,* emotional demands*,* hiding emotions*,* role conflicts*,* quality of leadership*,* support at work*,* unfair treatment*,* feedback*,* and sense of community)* with *commitment* and *job satisfaction* were determined. Multicollinearity among predictors was evaluated using the Variance Inflation Factor (VIF). Full models, including all independent factors, were calculated. Then, backward selection was performed manually by removing the factor with the highest p-value. If the adjusted R-square increased, the model was further reduced. The residuals of the final models were visually checked for normality. Moreover, homoscedasticity was tested using the Breusch-Pagan test. If this test was positive, robust standard errors were reported.

## Results

### Participants

**Fig. 1:**
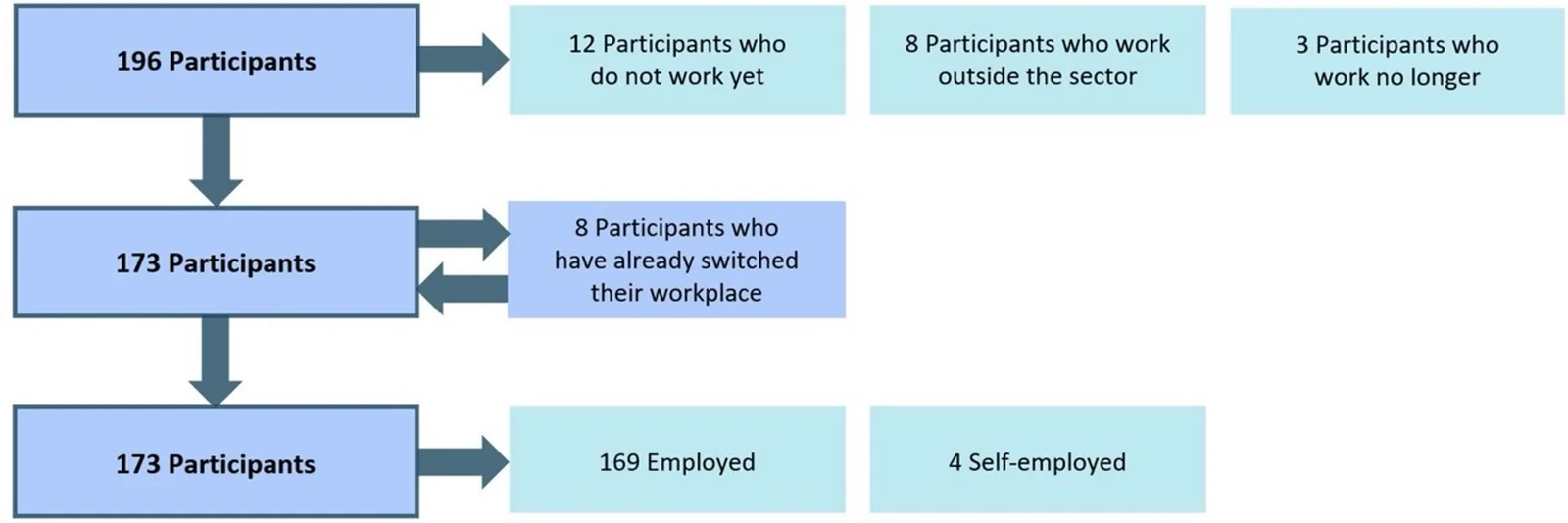
Employment of career starters up to 12 months after completing veterinary studies in a survey on early-career veterinarians in Germany, Austria, and Switzerland (2024)

The survey was opened by a total of 314 people who answered at least the first question. During data cleaning, persons who completed only 1 page, had duplicate records, graduated before 2023, or had been employed for > 12 months were excluded (*n* = 118). In the end, the responses of 196 participants were evaluated. In 2023, there were 933 graduates in veterinary medicine in Germany [34]. With 184 respondents from Germany, this corresponds to a response rate of 20% regarding the German participation. However, participation was lower in Austria and Switzerland with only 11 respondents. The study was originally designed for graduates from Germany and was later expanded to include Austria and Switzerland. Since admission to veterinary programs in Germany is regulated and the number of graduates varies only slightly from year to year, the total number of graduates for the year 2023 from all German veterinary schools was available and was used as the benchmark for calculating the response rate. Figure 1 illustrates the number of respondents who worked in veterinary medicine, whether they had already changed jobs, and whether they were employed or self-employed. In total, 173 respondents work in veterinary medicine, of whom 5% had already changed jobs. It is worth noting here that 5% had already changed employers at a very early stage in their careers. Such early job changes could indicate difficulties in the transition to clinical practice or a lack of compatibility between the graduates and their first employer. Most respondents (91%) graduated in 2024, and 7% graduated in 2023. The remaining 2% of respondents graduated between 2015 and 2019, but reported working for less than 12 months as veterinarians. The mean duration of the actual employment was 4 months (median).

### Demographics and work characteristics

The proportion of women roughly matches that of graduates in Germany (86%; 35). Most respondents (45%) completed their studies at the University of Munich, followed by the University of Hanover (17%) and the Freie Universität Berlin (12%). Graduates from other universities accounted for less than 10% each. Around 76% of young veterinarians worked in clinical practice. This proportion is slightly higher than the proportion of veterinarians who were working in clinical practice in Germany (68%; 35). Most respondents worked mainly with small animals (Table 2).

**Table 2:** Demographic information of early-career veterinarians from a survey in Germany, Austria, and Switzerland (2024)

	Respondents	Proportion (%)
Gender (*n* = 195)		
Female	171	87.7
Male	23	11.8
Diverse	1	0.5
Universities (*n* = 195)		
Freie Universität Berlin	23	11.8
University of Veterinary Medicine Hannover, Foundation	34	17.4
Leipzig University	19	9.7
Justus Liebig University Gießen	21	10.8
Ludwig-Maximilians-Universität München	87	44.6
Universities outside of Germany	11	5.6
Area of work (*n* = 156)		
Curative medicine	118	75.6
Non-curative medicine	38	24.4
Animal species (only 118 curative working veterinarians)		
Small animals	61	51.7
Ruminants	30	25.4
Horses	20	16.9
Other animal species	7	5.9
Care for other persons (children, parents) (*n* = 156)		
Yes	6	3.5
No	164	96.5
Relocation – last 6 months (*n* = 170)		
Yes (> 50 km)	64	37.6
Yes (< 50 km)	25	14.7
No	81	47.6
Debts (*n* = 170)		
Yes	50	29.4
No	120	70.6

Table 3 presents the respondents’ working conditions. Around 50% of those in employment worked more than 40 h per week (incl. overtime). Approximately 60% worked up to 10 h of overtime per week. Overall, 68% of respondents were either currently involved in out of hours emergency duties (i.e., emergency care provided outside regular opening hours, which in Germany is a routine obligation for most practices rather than a distinct specialty setting) or expected to perform them in the future. Regarding onboarding, 66% of participants did not have a structured onboarding plan. However, 65% reported having a veterinary supervisor at the start of their career. Around 40% of respondents were writing a doctoral thesis. In Germany, Austria, and Switzerland, veterinarians have the option of writing a doctoral dissertation after completing their studies; however, this is not a mandatory requirement for clinical practice. 

**Table 3:** Working conditions of early-career veterinarians from a survey in Germany, Austria, and Switzerland (2024)

Working time per week (incl. overtime) (*n* = 152)	Respondent	Valid percentage of respondents
< 40 h	46	30.3
40 h	26	17.1
> 40 h	80	52.6
Overtime hours per week (*n* = 145)		
No overtime	29	20
< 10 h	90	62.1
10–20 h	19	13.1
> 20 h	7	4.8
Emergency service (*n* = 156)		
Active in emergency service	78	50.3
Planned after training	27	17.4
Not planned	50	32.3
Previous practical experience in the same professional field (*n* = 170)		
Yes	77	45.3
No	93	54.7
Probationary period (*n* = 156)		
Yes	87	55.8
No	69	44.2
Fixed-term employment contract (*n* = 153)		
Yes temporary	66	43.1
No unlimited	87	56.9
Onboarding plan (*n* = 156)		
Yes	53	34
No	103	66
Supervising veterinarian (*n* = 155)		
Yes	100	64.5
No	55	35.5
Dissertation (*n* = 169)		
In the context of my employment	32	18.9
In addition to my veterinary work	37	21.9
No	100	59.2

### Demands

**Fig. 2:**
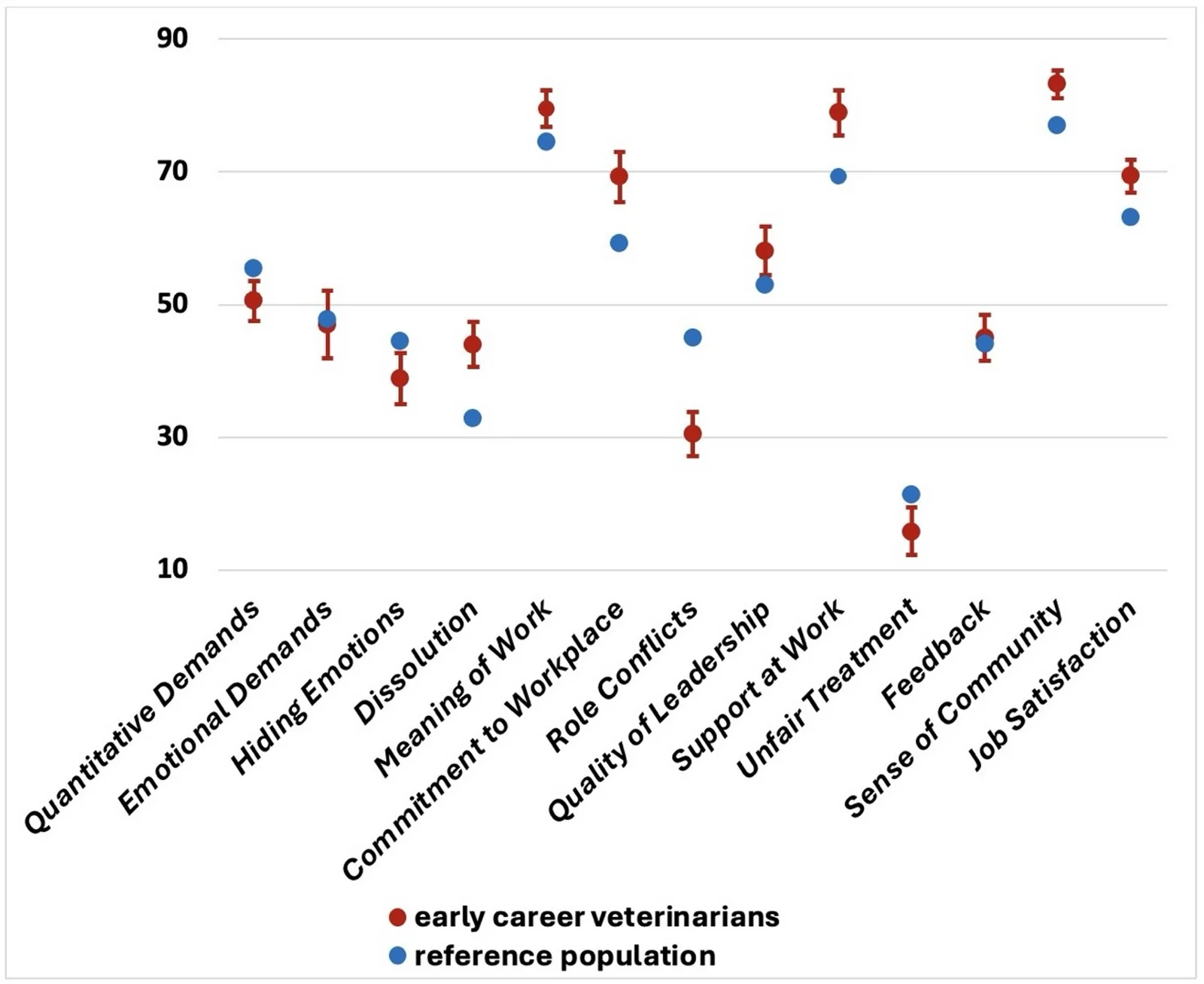
Means with 95% confidence intervals of scores for psychosocial dimensions from a survey (2024) of early-career veterinarians in Germany, Austria, and Switzerland (*n* = 196) compared to the reference population of German employees in various professions (27; *n* = 257,236)

The descriptive results of the dimensions are listed in Table 4. The comparison with the reference population is shown graphically in Fig. 2. Respondents of this survey reported significantly lower levels regarding *quantitative demands*,* role conflicts*, and *unfair treatment*. Means of *meaning of work*,* commitment to workplace*,* job satisfaction*,* quality of leadership*,* support at work*, and *sense of community*, but also *dissolution* was statistically significantly higher than in the reference population. Regarding *emotional demands* and *feedback*, no differences were observed. Cronbach´s Alpha was unacceptable for *dissolution* and *feedback.* For nearly all other dimensions, it was acceptable to good, except for emotional demands, which showed poor internal consistency [36]. The low internal consistency of the dissolution scale (Cronbach´s Alpha = 0.3) is reflected in the differing responses to its two items. While participants reported a comparatively high availability during their leisure time (mean = 52.4, SD = 29.0), they less frequently reported performing work related tasks outside regular working hours (mean = 35.1, SD = 27.1). Likewise, the feedback dimension (Cronbach´s Alpha = 0.4) combined ratings of feedback from supervisors (mean = 42.1, SD = 29.1) and colleagues (mean = 48.3, SD = 23.9), indicating that respondents differentiated between these two sources of feedback.

**Table 4:** Descriptive analyses of the psychosocial dimensions of early-career veterinarians within the first year using COPSOQ III [27] from a survey in Germany (2024)

Dimension	Items	Positive Value	*n*	Mean	SD	Cronbach´s Alpha
Quantitative Demands	QD1, QD2, QD3, QD4, QD5	low	146	50.5	18.2	0.8
Emotional Demands	ED6, ED7	low	149	47.0	31.7	0.6
Hiding Emotions	HE8, HE9	low	145	38.8	23.7	0.7
Dissolution	D5, D6	low	148	44.0	21.1	0.3*
Meaning of Work	MW3, MW4	high	151	79.6	17.0	0.8
Commitment to Workplace	CW5, CW6	high	149	69.2	23.6	0.8
Role Conflicts	RC6, RC7, RC8	low	142	30.5	20.3	0.8
Quality of Leadership	QL1, QL2, QL3, QL4	high	142	58.1	22.0	0.9
Support at Work	SW1, SW2, SW3, SW4	high	140	78.8	20.3	0.8
Unfair Treatment	UT10	low	147	15.8	22.1	-
Feedback	F5, F6	high	144	45.0	20.7	0.4*
Sense of Community	SC8, SC9	high	149	83.2	13.1	0.7
Job Satisfaction	JS1, JS2, JS3, JS4, JS5, JS6, JS7	high	146	69.4	14.9	0.8

* Cronbach´s Alpha < 0.60 interpret with caution because of low internal consistency

### Associations among demographics and work characteristics, and *commitment* and *job satisfaction*

The regression analyses examined the associations between demographic characteristics and *commitment* and *job satisfaction* (Table 5). It is particularly striking that measurable external factors, such as working hours, fixed-term contracts, or financial debts, have only a limited association with the target variables examined. In contrast, the presence of a supervising veterinarian was strongly associated with the two dependent variables.

**Table 5:** Results of multifactorial regression modeling exploring the association of sociodemographic and workplace characteristics with *commitment to workplace* and *job satisfaction* from a survey (2024), including early-career veterinarians from Germany, Austria, and Switzerland (*n* = 146)

Adj. *R* Square		Commitment to workplace**		Job Satisfaction**	
		0.145		0.148	
		estimate	*p*-value	estimate	*p*-value
Intercept		59.7 [6.3]	< 0.001	69.0 [2.9]	< 0.001
Gender	female vs. male	5.3 [5.3]	0.314	excl.*	excl.*
House move – last 6 months	yes (< 50 km) vs. no	excl.*	excl.*	6.3 [3.4]	0.069
	yes (> 50 km) vs. no	excl.*	excl.*	2.2 [2.6]	0.406
Previous practical experience in the same professional field	yes vs. no	2.9 [4.2]	0.494	excl.*	excl.*
Dissertation	yes vs. no	excl.*	excl.*	excl.*	excl.*
Curative activity	yes vs. no	-10.1 [7.0]	0.150	excl.*	excl.*
Emergency service	yes vs. no	-6.5 [3.9]	0.099	-5.1 [2.4]	0.034
Fixed-term employment contract	yes vs. no	-5.4 [3.9]	0.170	-2.8 [2.4]	0.254
Supervising veterinarian	yes vs. no	17.9 [4.5]	< 0.001	11.4 [2.7]	< 0.001
Debts	yes vs. no	excl.*	excl.*	excl.*	excl.*
Duration of current activity in months	Since the beginning of the current activity	1.2 [0.9]	0.166	excl.*	excl.*
Overtime hours per week	Gap between actual and contractual hours	excl.*	excl.*	excl.*	excl.*
Probationary period	yes vs. no	excl.*	excl.*	excl.*	excl.*
Induction plan	yes vs. no	excl.*	excl.*	excl.*	excl.*

*Excluded; not in the final model

**Robust standard error as homoscedasticity was not given

### Associations among demands and commitment and job satisfaction

The results in Table 6 show the associations between the other COPSOQ dimensions and *commitment* and *job satisfaction*. *Commitment to workplace* was associated with *support at work*, *unfair treatment*, and *feedback*. *Job satisfaction* was positively associated with *leadership quality* and *feedback*. *Unfair treatment* was negatively associated with *job satisfaction* and *commitment to the workplace*.

**Table 6:** Difference in *commitment to workplace* and *job satisfaction* according to selected dimensions of the COPSOQ: *Quantitative demands*,* emotional demands*,* hiding emotions*,* role conflicts*,* quality of leadership*,* support at work*,* unfair treatment*,* feedback*, and *sense of community* of 146 early-career veterinarians

Adj. *R*-Square	Commitment to workplace		Job Satisfaction	
	0.427		0.549	
	estimate	*p*-value	estimate	*p*-value
Intercept	31.6 [9.9]	0.002	46.2 [7.9]	< 0.001
Quantitative Demands	excl.*	excl.*	-0.1 [0.1]	0.102
Emotional Demands	excl.*	excl.*	0.04 [0.1]	0.357
Hiding Emotions	excl.*	excl.*	-0.1 [0.1]	0.044
Role Conflicts	-0.1 [0.1]	0.332	excl.*	excl.*
Quality of Leadership	0.1 [0.1]	0.287	0.2 [0.1]	0.002
Support at Work	0.3 [0.1]	0.004	0.1 [0.1]	0.239
Unfair Treatment	-0.3 [0.1]	0.001	-0.1 [0.1]	0.006
Feedback	0.3 [0.1]	< 0.001	0.2 [0.1]	< 0.001
Sense of Community	excl.*	excl.*	0.1 [0.1]	0.235

*Excluded; not in the final model

## Discussion

This is one of the first studies to explore job satisfaction and commitment to the workplace among early-career veterinarians using a validated assessment tool (COPSOQ). The results suggest that, contrary to previous research, *job satisfaction* was generally high and *quantitative demands* were lower compared to the COPSOQ reference population.

### Workplace characteristics

The results showed that most of the young veterinarians worked in clinical practice. Within clinical practice, the main focus is on small animals, followed by ruminants and horses. This distribution largely aligns with the preferences of German graduates in 2023, confirming the trend toward dominance of small-animal practice among young professionals [37].

The description of workplace characteristics showed that most respondents were highly engaged, as they worked more than 40 h per week, participated in emergency services or planned to do so, and worked extra hours. This confirms earlier studies that pointed to a high workload in the first years of employment [12, 38].

### Psychosocial demands and *job satisfaction*

Contrary to our main hypothesis that levels of demands, *dissolution* of boundaries and *role conflicts* are higher in early-career veterinarians when compared to other professions, early-career veterinarians were less demanded and more satisfied than the German reference population of the COPSOQ III validation study [28], and also when compared to a population of official veterinarians in Germany [39]. Previous studies compared the job satisfaction and stress levels of early career veterinarians to veterinarians with a higher level of job experience. Those studies showed that early-career veterinarians are in particular demanded. This study took a different approach and examined job satisfaction of early-career veterinarians with the general German reference population of the COPSOQ III. The results indicate higher job satisfaction and lower psychosocial demands relative to this general German reference population. No conclusions can be drawn regarding the stress levels or job satisfaction of more experienced veterinarians. Similarly, no conclusions can be drawn regarding mental health conditions. Regarding levels of burnout, anxiety and depressions, results will presented in a second manuscript [1, 11, 20]. However, in a recent study, higher job satisfaction was observed among younger veterinarians than among their older colleagues [18]. When starting a new position, *job satisfaction* might reach a peak after organizational entry (a period termed the “honeymoon phase”) and decreases thereafter (“hangover”). In addition, having recently completed veterinary school may contribute to this initial increase in job satisfaction during the transition to professional practice [40]. However, in a study exploring this pattern among persons without prior job experience, the hangover effect was not observed among a subgroup who remained satisfied [41]. The analysis of follow-ups after six and twelve months in our study will provide further insights into the development of *job satisfaction* and the other scales. Maybe job satisfaction will decrease, and psychosocial demands might increase after the first year in the job, when veterinarians take on more responsibility at work. This observation may also be attributable to a waning honeymoon effect. Up to now, months within the workplace have had no statistically significant association with *job satisfaction* or *commitment*. Therefore, the reasons for the higher *job satisfaction* observed relative to the general German COPSOQ reference population might include better working conditions than some years ago, more support from supervisors or colleagues, the meaningfulness of the profession, or a better financial situation than before, when participants were still studying.

Another surprising finding was the low incidence of role conflicts. During their training, veterinarians often learn “best practices,” for example in the area of intensive diagnostics. Upon entering the “real world,” we had expected role conflicts to arise, as they work with clients facing financial constraints and are unable to offer the gold standard of care [24]. One possible explanation for this is that newly licensed veterinarians may initially have only a limited degree of responsibility, since many decisions are made or co-decided by more experienced colleagues. It is also conceivable that a supportive work environment and structured mentorship during the transition to private practice mitigate potential role conflicts. Furthermore, we had expected a lower rating of leadership quality, as previous studies had shown large differences in the perception of leadership [39, 42, 43].

The high levels of *commitment* and *dissolution* relative to the general German COPSOQ reference population were not surprising. Another study found that veterinarians, in their efforts to provide the best possible care for animals and their owners, often push themselves beyond their limits in terms of working hours, physical endurance, and mental resilience [44]. On one hand, high dissolution might be a sign that veterinarians see a job as a calling in terms of meaningfulness and compassion satisfaction [2, 3]. On the other hand, high dissolution can lead to stress or sleeping disorders [8]. Furthermore, a study found that people who think about work in their free time show a significantly increased risk of depression and suicide [9]. Therefore, early-career veterinarians should be trained in terms of boundary management and coping strategies to find a level of *dissolution* that contributes to long-term mental health.

### Associations among demographics, work characteristics, psychosocial demands and *commitment* and *job satisfaction*

Contrary to expectations, emergency services and overtime were not associated with *commitment to the workplace* or *job satisfaction*. This statement is not in line with previous research showing that on-call duties and participation in emergency services negatively influenced *job satisfaction* [6, 43]. In fact, the only factor that showed a statistically significant association with the three dependent variables in the study presented here was the availability of a supervising veterinarian. People who reported having a supervisor showed higher *job satisfaction* and greater *commitment to workplace.* Other studies have found similar results: Graduating veterinary students identified good mentorship as the most important non-monetary feature of employment [45]. A study in the Netherlands found that many professionals left their jobs within the first five years due to negative experiences. One of the main reasons for this was a lack of support from employers [46]. In addition, the study by Jansen et al. (2024) showed that the early stages of a career are particularly challenged by taking on responsibility without support [47]. Also, a study of veterinarians who pursued an academic career found that management support is crucial for retaining both new entrants and assistant physicians in this field [38]. Therefore, it can be concluded that a supervising veterinarian for early-career veterinarians is not a nice-to-have, but an essential part of onboarding.

Targeted measures to promote structural support - such as onboarding and mentoring programs - can help ease the transition into the profession, increase satisfaction, and strengthen long-term commitment. Therefore, we suggest enhancing leadership training as part of the lifelong learning for veterinarians. Further research is needed on the progression of *job satisfaction* and psychosocial stress, especially given the increasing evidence that veterinarians leave the profession in their third or fourth year of practice [46], suggesting that *job satisfaction* declines after a certain period of time. Studies from other countries are needed to address whether our generally satisfied and engaged cohort of early-career veterinarians is an exception or a future trend towards improvement in the veterinary profession.

Regarding the association between different psychosocial dimensions and job satisfaction and commitment, support, quality of leadership, and feedback played a major role, while emotional demands were of minor importance. As expected, quantitative demands enhanced *dissolution*, and the *hiding of emotions* decreased *job satisfaction*. This association is consistent with a previous study showing that hiding of emotions was associated with distress [9].

### Limitations

Like in other surveys with voluntary participation, a response bias cannot be ruled out. People with an already heavy workload may not have participated in the survey. This would have led to an underestimation of demands and an overestimation of *job satisfaction*. On the other hand, a bias in the other direction might also exist. People who have had negative experiences at the start of their careers might have been more motivated to share their experiences. Moreover, the findings of this observational study should be interpreted in the context of its specific setting. The data reflect conditions in the included countries, primarily Germany, during the study period and may not be generalizable to other regions or time periods. Consequently, caution is warranted when extrapolating these results beyond the populations and conditions examined in this study.

Given the cross-sectional design of this study, causal inferences cannot be drawn. Working conditions and psychosocial demands were assessed simultaneously. Moreover, as the transition into the veterinary profession can only be examined in real-world contexts rather than under well-controlled conditions, this field-based approach inherently limits the option to control for confounding by study design. It does not allow for definitive causal claims.

The distribution of genders reflects the gender ratio in veterinary medicine [35]. Respondents from the university in Munich were overrepresented. This is likely because the first author studied there and was part of the target population. Therefore, former fellow students might have been more motivated because they knew the person who invited them to participate. Graduates from universities outside Germany were underrepresented; as a result, the results mainly reflect the situation of veterinary graduates in Germany.

When comparing our population to the German reference population, it should be kept in mind that the two populations differ not only concerning their workplace but also concerning demographic characteristics like gender and age. The reference population was derived from 966 surveys in all kinds of companies throughout Germany. About half of the respondents were female and only 30% were younger than 35 years [28]. Young and female employees tend to have a higher job satisfaction than middle-aged and male employees [48, 49]. However, these trends are not universal and in the validation study of the German COPSOQ, gender and age were not significantly associated with job satisfaction [28]. So, only minor bias is expected to demographic differences between the two populations. Moreover, this comparison makes it possible for the first time to classify the psychosocial stress of early-career veterinarians.

Even though most students graduated in early spring, they have entered the profession at different dates. Moreover, graduates from 2023 or earlier have been included, as they fall within the target population. This led to some participants having just begun their work, while others had been working as veterinarians for nearly 1 year. As working conditions and satisfaction might change over time, the months spent in the actual workplace were included in the model, but were not significantly associated with *commitment* or *job satisfaction*.

In general, COPSOQ III is a recognized and validated instrument for assessing various dimensions of psychosocial working conditions [28]. However, since COPSOQ III is not a standardized, ready-to-use instrument, the questionnaires used may differ. Therefore, caution is required when comparing our results to other surveys, as dimensions with the same designation may include different items. To ensure comparability, the dimensions were calculated according to Lincke et al. (2021), and the selection of the respective items was based on the population of that validation study [28]. Two dimensions showed an unacceptable low internal consistency (Table 4): *dissolution* and *feedback*. Both dimensions consist of only two items, which appear to assess different aspects. In the international standard version, the dimension *dissolution* is not included. In the German version, it has the two items: (i) “I take care of work-related tasks outside of my working time as well,” and (ii) “I‘m available in my free time for people with whom I deal professionally.” [33]. Both aspects concern the blurring of professional and private life. However, people who are often available for people they work with in their free time are not necessarily taking care of work tasks during that time, and vice versa. Therefore, *dissolution* must be regarded with caution. Regarding the dimension *feedback*, both items refer to feedback from different people (the supervisor and colleagues), and the assessments might differ among them. Here, the mean can be interpreted as an “overall” feedback assessment, since missing feedback from one person might be offset by another person’s feedback.

## Conclusion

This first survey on young veterinarians starting their careers shows that, despite a sometimes heavy workload, high *job satisfaction* and *commitment* can be achieved if supportive conditions are in place. In particular, the findings highlight the importance of supervision and mentorship during the transition into professional practice, emphasizing the role of experienced colleagues in fostering a positive work environment and successful career entry.

Finally, future research should broaden the focus beyond the association among working conditions, job satisfaction, and psychosocial demands to include mental health and psychological capital as relevant outcomes and resources. Such an integrative perspective would acknowledge that early-career development is shaped not only by good day-one competencies and external work-related circumstances, but also by individual psychological factors. Considering both structural conditions and personal resources may therefore provide a more comprehensive understanding of well-being during the transition from student to a motivated and happy veterinarian.

## Supplementary Information

Supplementary Material 1: Questionnaire. Self-developed questionnaire in the original language and translated to English.

## Data Availability

The datasets generated and analysed during the current study are not publicly available due to data protection regulations but might be made available from the corresponding author on reasonable request.

## Abbreviations

COPSOQ: Copenhagen Psychosocial Questionnaire
PHQ-4: Patient Health Questionnaire-4
OLBI: Oldenburg Burnout Inventory
PSI-16: Psychological Capital Inventory
FCS: Fully conditional specification
VIF: Variance Inflation Factor
