# Supplementary material for: Part I of “Navigating the transition”: early-career working conditions, psychosocial demands, and job satisfaction among German, Austrian, and Swiss veterinarians – a first-year survey
Source: BMC Vet Res. 2026 Sep 21;22:553. doi: 10.1186/s12917-026-05923-9 (PMC13599086; doi:10.1186/s12917-026-05923-9)
Supplement: Supplementary file 1 [file 12917_2026_5923_MOESM1_ESM.pdf]

Starting a Career in Veterinary Medicine

Dear colleagues,

My name is Amelie Wetter. I am a newly licensed veterinarian and recently started my first professional position. I am currently writing my doctoral thesis at the Institute of Veterinary Epidemiology and Biometry at Freie Universität Berlin.

As part of my doctoral research, we would like to identify the challenges associated with entering the veterinary profession. Based on the results, recommendations can be developed to facilitate the transition into professional veterinary work.

We would greatly appreciate it if you could take a few minutes to participate in the survey. Completing the questionnaire takes approximately 15–20 minutes.

For the success of the study, it is important that you complete the questionnaire as fully as possible. Please do not be surprised if many questions and statements sound similar or seem unrelated to veterinary work. These are question blocks from established social science measurement instruments that allow us to compare the results with those of other professions.

All data will be collected anonymously and cannot be linked to your person. After submitting the survey, you will have the opportunity to provide an email address via a separate link so that we can invite you to a follow-up survey in a few months and share the study results with you.

Survey Code:

To track developments over time while maintaining anonymity, please create a survey code using the following:

- Second letter of your first name
- Day of birth (two digits)
- Second letter of your surname
- First letter of your place of birth

Demographic Information:

Which gender do you identify with?

- Female
- Male
- Diverse

In which year were you born?

Where did you mainly grow up?

- Germany
- Other European country
- Outside Europe

In which year did you receive your veterinary license?

At which university did you obtain your veterinary degree?

- Berlin
- Hanover
- Leipzig
- Giessen
- Munich
- Vienna
- Zurich
- Bern
- Budapest
- Other

Are you currently employed?

- Yes, mainly in veterinary medicine
- Yes, mainly outside veterinary medicine
- Yes, but currently on maternity leave, parental leave, or long-term sick leave
- No, I was employed but currently am not
- No, I have not yet been employed since graduation

Where do you currently work?

- Austria
- Switzerland
- Baden-Württemberg
- Bavaria
- Berlin
- Brandenburg
- Bremen
- Hamburg
- Hesse
- Mecklenburg-Western Pomerania
- Lower Saxony
- North Rhine-Westphalia
- Rhineland-Palatinate
- Saarland
- Saxony-Anhalt
- Saxony
- Schleswig-Holstein
- Thuringia
- Other

Are you employed or self-employed?
- Employed
- Self-employed (new business)
- Self-employed (joining an existing company)

Have you moved in the last six months?
- Yes, within the region (<50km)
- Yes, to another region (>50km)
- No

If move = yes:
Have you ever lived in this region before?
- Yes
- No

*If move = yes:*What was the main reason for your move?
- Occupation
- Family
- Partnership
- Other

Are you currently in a relationship?
- Yes
- No

Are you responsible for caring for people (minors or persons in need of care) in your household?

- Yes, one person
- Yes, several people
- No

Do you currently have any student loans (e.g., credit, Bafög)?

- Yes
- No

Do you find these debts burdensome?

- Yes very
- Yes something
- Rather not
- No

Are these debts or financial burdens resulting from veterinary studies?

- Yes
- No

Can you live financially independently of your parents/partner or other people in your environment?

- Yes, while maintaining the current lifestyle
- Yes, but I had to severely restrict myself
- No

Did you already have practical experience working in a veterinary field before graduating (excluding mandatory internships during your studies)?

- Yes, in a similar field to the one I am currently working in
- Yes, in a different field than the one I am currently working in
- No

How long have you been working in this field?

Are you currently working on a veterinary dissertation?

- Yes, as part of my employment relationship
- Yes, in addition to my veterinary work
- No

Are you currently in your first veterinary position after receiving your license to practice?

- Yes
- No, I changed jobs

What were the reasons for your change?

How long were you employed in your previous veterinary position?

How important were the following aspects in your decision to join your current employer?

	Very high importance	High importance	Low importance	No importance
Financial security				
Career potential				
Opportunities for further training / specialization				
Gaining professional experience				
Great team / good working atmosphere				
Recommendation from friends / acquaintances				
Recruitment and getting to know the workplace through internships				
Local accessibility of the workplace				
Interest in research				
Personal fulfillment				
Work-life balance				
Opportunity to work part-time				

Are there any other reasons that led you to start working for your current employer?

Do you primarily provide curative veterinary care?

- Yes
- No

If mainly curative:
Which animal species accounts for the largest portion of your veterinary practice?

- Pets (cats/ dogs)
- Ruminants
- Horses
- Pigs
- Poultry
- Small mammals
- Birds/exotic animals
- Other

If mainly curative:
What best describes the workplace where you practice veterinary medicine?

- University hospital
- Veterinary clinic
- Animal health center
- Specialized veterinary practice
- General veterinary practice
- Other

If mainly curative:
Is the veterinary practice where you work part of an investment group (e.g., Anicura, Evidensia, or TierarztPlusPartner)?

- Yes
- No

*If **not** mainly curative:*
In which area do you primarily work?

- ○ Research facility
- ○ Government agency/slaughterhouse
- ○ Industry (pharmaceutical industry, feed industry, etc.)
- ○ Other

In which area do you primarily work?

How many veterinary colleagues do you have at your current workplace?

- ○ 0
- ○ 1-2
- ○ 3-5
- ○ 6-9
- ○ 10-19
- ○ 20-29
- ○ 30-39
- ○ >39

When did you start working as a veterinarian for your current employer?

What are your contractually agreed working hours (in hours) per week?

How many hours do you actually work on average per week (including on-call duty, if applicable)?

Do you work in emergency service?

- ○ Yes, I work in emergency service
- ○ No, not yet, but it is planned after training

- No, and it is not planned in the long term either

If emergency service = yes:
In what form do you perform your emergency service?

- Regular duties during emergency hours
- On-call duty
- Standby duty

If emergency service = yes and regular duties = yes:
How many hours of regular duties do you perform during emergency hours each month?

If emergency service = yes and on-call duties = yes:
How many hours of on-call duty do you perform during emergency hours each month?

If emergency service = yes and standby duties = yes:
How many hours of standby duty do you perform during emergency hours each month?

If emergency service = yes:
How many other veterinarians work a shift in the emergency department at your workplace?

- No other veterinarians are normally on duty
- There is usually one other veterinarian on duty
- There are usually two or more veterinarians on duty

If emergency service = yes:

Is support staff (e.g., veterinary technicians, students) usually available on-site to assist with veterinary work during the emergency shift?

- ○ Yes
- ○ No

Is there a support team you can contact for questions or assistance?

- ○ Yes
- ○ No

Is your employment contract temporary?

- ○ Yes
- ○ No

If contract= temporary:
When does your employment contract end?

Are you currently still in your probationary period?

- ○ Yes
- ○ No

To what extent did/do the following aspects correspond to the agreements made at the time of hiring?

	Completely	Minor deviations	Significant deviations	Not at all	Not applicable
Working hours					
Salary					
Onboarding					
Animal species to be treated					

Length of the probationary period					

Are there any other agreements made at the time of hiring that no longer apply?

Is there a supervising veterinarian at your workplace who is responsible for your onboarding?

- ○ Yes
- ○ No

Does your workplace have an orientation plan?

- ○ Yes
- ○ No

How satisfied were/are you with your onboarding to veterinary tasks and procedures?

- ○ 1 (very good)
- ○ 2 (good)
- ○ 3 (satisfactory)
- ○ 4 (adequate)
- ○ 5 (poor)
- ○ 6 (unsatisfactory)

What are the reasons for your dissatisfaction with the orientation?

Followed by:

Items of the Copenhagenpsychosocial Questionnaire III

Oldenburg Burnout Inventory

Patient-Health-Questionnaire

Psychological Capital Inventory

At the End of the Survey

Thank you very much for participating in the survey.

If the questions caused emotional distress or if you notice signs of depression, burnout, or other mental health issues, please seek support from people around you and professional services.

Helpful resources include:
- General practitioners
- Telephone counseling service: 0800 1110111
- https://vetivolution.org/
- https://vethilfe.org/
- Psychiatric emergency services
- Support groups

Thank you very much for your participation.
